# Biochemical and Functional Characterization of *E. coli* Aminopeptidase N: A New Role as a 6-Monoacetylmorphine Hydrolase

**DOI:** 10.3390/biom15060822

**Published:** 2025-06-05

**Authors:** Xiabin Chen, Yishuang Li, Jianzhuang Yao, Xiaoxuan Li, Hualing Li, Zelin Wu, Qi Hu, Nuo Xu, Tingjun Hou, Jiye Wang, Shurong Hou

**Affiliations:** 1 School of Pharmacy, Hangzhou Normal University, Hangzhou 311121, China; 2 School of Biological Science and Technology, University of Jinan, Jinan 250022, China; 3 College of Pharmaceutical Sciences, Zhejiang University, Hangzhou 310058, China; 4 Key Laboratory of Drug Prevention and Control Technology of Zhejiang Province, Zhejiang Police College, Hangzhou 310053, China

**Keywords:** *Escherichia coli* aminopeptidase N, heroin and 6-MAM, biochemical property, catalytic function

## Abstract

6-monoacetylmorphine (6-MAM), a primary active metabolite of heroin that reaches the human brain, plays a crucial role in producing heroin-associated physiological and lethal effects. Therefore, 6-MAM has emerged as a key target for alleviating the adverse consequences of heroin abuse. In this study, the proposed 6-MAM hydrolase *E. coli* aminopeptidase N (eAPN) was recombinantly produced, and its biochemical and functional profiles were investigated. eAPN’s biochemical properties, with respect to pH, metal ions, and temperature, and catalytic functions toward peptidase substrates and 6-MAM were thoroughly examined. Extensive experiments reveal that incorporation of an N-terminal His-tag notably affects eAPN’s aminopeptidase activity. This cost-effective recombinant eAPN exhibits favorable thermostability and optimal activity at pH 7.5. Kinetic analysis toward peptidase substrates reveals that eAPN preferentially cleaves peptides following amino acid residues in the order of Ala > Arg >> Met, Gly > Leu > Pro, indicating a preference for small or basic amino acid residues as substrates. Computational and experimental studies have, for the first time, discovered that eAPN is capable of catalyzing the hydrolysis of heroin and 6-MAM, which has shed light on its functional versatility and potential applications. This work elucidates the biochemical properties of eAPN and expands its catalytic functions, thereby laying the groundwork for a deep understanding and further reengineering of eAPN to enhance its activity toward 6-MAM for heroin detoxification.

## 1. Introduction

Heroin (3,6-diacetylmorphine), synthesized from morphine alkaloids in 1874 [1], is a potent opioid receptor agonist that primarily targets μ receptors [2]. In the realm of medicine, heroin was originally employed as an analgesic and cough suppressant, due to its remarkable pain-relieving capabilities [3]. As an analgesic, heroin is 2–4 times more potent than morphine and exhibits a swift onset of action [4]. Nonetheless, the high potential for abuse and severe side effects associated with heroin use, including addiction, immune system deterioration, respiratory infections, and liver and kidney damage swiftly transformed it into a significant public health concern [5]. Consequently, heroin is now categorized as a strictly controlled substance in numerous countries and regions worldwide [6].

**Figure 1:**
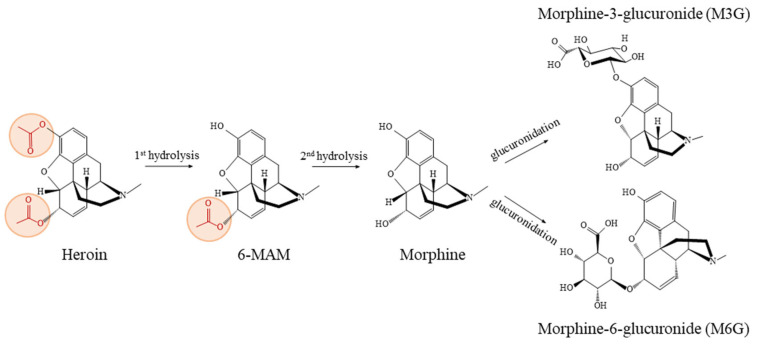
The biotransformation process of heroin and its metabolites in humans [3,7].

The half-life of heroin is remarkably brief, ranging from approximately 1.3 to 7.8 min [1]. The metabolism process of heroin and its metabolites [3,7] (Figure 1) encompasses several key reactions, as follows: (1) hydrolytic reactions, facilitated by serum butyrylcholinesterase (BChE), erythrocyte acetylcholinesterase (AChE), and carboxylesterases (CES) mainly in the liver, kidney, and intestine; (2) synthetic reactions, predominantly glucuronidation by UDP-glucuronosyltransferase (UGT) occurring primarily in the liver (with contributions also from the brain, kidney, and intestine), and to a lesser extent, sulfation also plays a role; (3) oxidative reactions, resulting in the formation of minor metabolites, such as N-demethylation via CYP3A4 and CYP2C8 isoenzymes producing normorphine [8]. The rate at which heroin and its metabolites transfer across the blood–brain barrier primarily depends on their lipophilicity, which undergoes a gradual reduction at each stage of metabolism, i.e., heroin > 6-MAM > morphine > M6G [3]. The structural characteristics of heroin confer it with high lipophilicity, enabling rapid conversion into the active metabolite 6-MAM in the body upon administration [7,9,10,11]. This metabolite is a key indicator of heroin use in urine. More importantly, 6-MAM is the primary active metabolite that reaches the brain, where it binds to μ-opioid receptors with high efficacy through its free hydroxyl phenolic group [12,13], thereby producing respiratory depressant activity [5]. Thus, 6-MAM emerges as a pivotal factor in producing the physiological and lethal effects associated with heroin use.

In recent years, enzyme therapy has received widespread attention and demonstrated great potential in the treatment of drug abuse, particularly in the realm of cocaine overdose and addiction [14,15,16]. Enzymes can efficiently catalyze the breakdown or conversion of drugs into desired products, thereby accelerating drug metabolism and elimination from the body. Additionally, their safety is normally ensured, as they act directly on drug molecules with great affinity and specificity and do not cross the blood–brain barrier, reducing the risk of producing side effects or toxicity in central nervous system [16,17]. Enzyme therapy has gained prominence in drug abuse treatment due to its exceptional efficacy and safety features, which have been proved in clinical studies [15,16,17]. These features make enzymes highly specific and potent, enabling them to perform therapeutic biochemistry in the body that small molecules cannot achieve. Consequently, enzyme therapy offers superiority in efficacy, precision, and safety for the treatment of such types of disorders [18,19].

Considering the significant role of 6-MAM in producing heroin-associated harmful effects, it is of great significance to discover and/or develop enzymes to accelerate its metabolism. Although the abovementioned esterases, including AChE, BChE, CES1, and CES2, can hydrolyze both heroin and 6-MAM, they possess relatively low catalytic efficiencies toward 6-MAM [20,21,22]. It is not sufficient to achieve quick degradation for clinical application. Therefore, identifying and discovering enzymes with greater catalytic efficiency for 6-MAM hydrolysis holds paramount significance for the treatment of 6-MAM toxicity. Computational screening based on key catalytic residues has identified a potential 6-MAM hydrolase, *E. coli* aminopeptidase N (eAPN). eAPN belongs to the M1 family of peptidases [23] and was recognized as the sole alanine aminopeptidase in *E. coli* [24]. Through recombinant expression, purification, and kinetic analysis, eAPN is confirmed to exhibit catalytic activity toward heroin and 6-MAM, with their kinetic parameters (*k*_cat_ and *K*_M_) characterized. Additionally, this study also examined its biochemical and functional profiles, with respect to pH, metal ions, and temperature, as well as its substrate specificity for peptidase substrates. This investigation enhances our understanding of the biochemical properties and catalytic functions of eAPN and provides guidance for the further engineering of eAPN, which holds potential application in heroin detoxification.

## 2. Materials and Methods

### 2.1. Materials

*E. coli* DH5α cells were sourced from Tsingke Biological Technology (Beijing, China). The FastPure EndoFree Plasmid Maxi Kit was from Vazyme Biotech (Nanjing, China). The BCA Protein Assay Kit was purchased from Beyotime Biotechnology (Shanghai, China). HisSep Ni-NTA agarose resin for affinity chromatography was from Yeasen Biotechnology (Shanghai, China). Anti-His mouse monoclonal antibody was obtained from Affinity Biosciences (Cincinnati, OH, USA). Amicon Ultra-50 kD centrifugal filters were purchased from Millipore (Burlington, MA, USA). Heroin, 6-MAM, and morphine were obtained from Shanghai Yuansi Standard Science and Technology (Shanghai, China). L-Ala-*p*-nitroanilide, L-Leu-*p*-nitroanilide, L-Pro-*p*-nitroanilide, L-Met-*p*-nitroanilide, L-Gly-*p*-nitroanilide, and L-Arg-*p*-nitroanilide were sourced from Aladdin Biochemical Technology (Shanghai, China). Thrombin was obtained from Yuanye Biotechnology (Shanghai, China). All other chemicals including the solvents used in high-performance liquid chromatography (HPLC) were purchased from Shanghai Macklin Biochemical Technology (Shanghai, China). SYPRO^®^ Orange dye was obtained from Sigma-Aldrich (St. Louis, MO, USA).

### 2.2. Protein Multiple Sequence Alignment and Phylogenetic Tree Analysis

To elucidate the evolutionary relationships and conservation patterns among the target protein and its homologs, multiple sequence alignment and phylogenetic tree analyses were conducted using well-established bioinformatics tools. The BLASTp program was employed to identify the members of M1 family of aminopeptidases that show high similarity with eAPN. Top hits across different species exhibiting an E value between 0 and 1 × 10^−8^ and the well-studied members were selected for sequence alignment and phylogenetic analyses. The protein sequences of selected members were retrieved from the UniProt database based on their homology to the target protein. Multiple sequence alignment was performed using ESPript to highlight conserved regions and identify potential functional motifs. The phylogenetic tree was generated using the neighbor-joining (NJ) method as implemented in MEGA 7.0 software. The alignment was subjected to 1000 bootstrap replicates to evaluate the robustness of the tree topology. The resulting tree was refined for clarity, with adjustments made to branch lengths and node labels. This analysis provides insights into the evolutionary relationships among the aligned sequences, revealing distinct clades and conserved lineages.

### 2.3. Construction of eAPN Plasmid

The cDNA (NCBI NP-415452.1) encoding eAPN was synthesized by Tsingke Biological Technology (Beijing, China) and cloned into a bacterial expression vector pET28a. This insertion included an N-terminal 6×His tag and a thrombin cleavage site (MGSSHHHHHHSSGLVPR↓GS-eAPN). The plasmid was then transformed into *E. coli* DH5α competent cells, and the transformed cells were plated on LB agar plates supplemented with 50 μg/mL kanamycin. Finally, the plasmid was amplified, extracted, and purified using the FastPure EndoFree Plasmid Maxi Kit.

### 2.4. Over-Expression and Purification of Recombinant eAPN

An overnight pre-culture of *E. coli* BL21 cells transformed with the pET28a-eAPN plasmids was used to inoculate 1 L of LB medium supplemented with 50 μg/mL kanamycin. The cells were shaken and cultured at 37 °C until they reached an optical density (OD_600nm_) of 0.6–0.8. The expression of the enzyme was induced with 1 mM isopropylthio-β-galactoside (IPTG), and cells were shaken at 17 °C for an additional 16–18 h. The cells were collected by centrifugation for 15 min at 4000 rpm and then resuspended in 5 mL of cold binding buffer consisting of 50 mM Tris-HCl, pH 7.5, and 100 mM NaCl.

The resuspended cells were lysed with a French press (Scientz, Ningbo, China). The enzyme was then purified by Ni-NTA affinity chromatography. After binding with the resin for 2 h at 4 °C, the sample was loaded into the column. Non-target proteins were removed by washing with the binding buffer supplemented with 10 mM imidazole. The target protein was eluted using the binding buffer containing 30 mM imidazole. To remove the His-tag from the expressed protein (denoted as His-eAPN), thrombin (20 U, ~10 µg) was incubated with His-eAPN (10 mg) in 20 mM Tris-HCl, pH 8.0, and 150 mM NaCl at 4 °C for 16 h. Subsequently, Ni-NTA affinity chromatography was used to separate eAPN and residual His-eAPN. eAPN along with thrombin was collected from the flow-through and dialyzed with Amicon Ultra-50 kD centrifugal filters to eliminate thrombin from the mixture. eAPN was concentrated, aliquoted, and stored at −80 °C in the binding buffer supplemented with 20% glycerol. To conduct subsequent experiments, the enzyme concentration was accurately determined using an Enhanced BCA Protein Assay Kit. The molecular weight and purity of the protein were verified through SDS-PAGE and Western blot analysis, and the multimeric nature of eAPN was assessed by Native PAGE.

### 2.5. Effect of pH, Thiol Reducer, Metal Ions

To explore the effect of pH on the enzyme activity of eAPN, its catalytic activity toward 1 mM L-Ala-*p*-nitroanilide was assayed at 37 °C across a pH range from 5.0 to 10.0. The buffers used in this study were 50 mM acetate buffer (pH 5.0–5.5), 50 mM phosphate buffer (pH 6.0–8.0), 50 mM Tris-HCl buffer (pH 8.5–9.0), and 50 mM glycine-NaOH buffer (9.5–10.0). The final enzyme concentrations were 0.5 nM for eAPN and 5 nM for His-eAPN, respectively. These concentrations were also employed in the subsequent assays detailed in Section 2.5. The method for determining aminopeptidase activity by monitoring the absorbance at 405 nm using a Microplate Reader (Tecan Spark, Männedorf, Switzerland) was as described in a previous literature [25]. The initial reaction rates were assessed from the linear part of the reaction curves and converted using the standard curve of reaction product *p*-nitroanilide.

Similarly, to determine the effect of thiol reducing agents on enzyme activity, the enzyme was incubated at 37 °C for 30 min in 50 mM phosphate buffer (pH 7.5), in the absence or presence of 1 mM tris-(2-carboxyethyl)phosphine (TCEP). Subsequently, enzyme activity was assayed as described above at pH 7.5 using 1 mM L-Ala-*p*-nitroanilide as the substrate.

The impact of various metal ions on the activity of eAPN was investigated by incubating the enzyme with different concentrations of metal ions in 50 mM phosphate buffer (pH 7.5), including KCl, LiCl, NiCl_2_, CoCl_2_, CuCl_2_, ZnCl_2_, MgCl_2_, CaCl_2_, MnCl_2_, and FeCl_3_ at 50 μM, 500 μM, and 2000 μM for 30 min, and their enzyme activities were determined accordingly under the standard conditions outlined above. The dose response of ZnCl_2_ was determined to confirm its effect on eAPN activity.

### 2.6. Effect of Temperature and Thermostability of eAPN

To evaluate the effect of temperature on eAPN activity, the enzyme (final 5 nM for eAPN, 50 nM for His-eAPN) was incubated in 50 mM phosphate buffer (pH 7.5), exposed to temperature in the range of 25–60 °C for 30 min, followed by a reaction at pH 7.5 with 1 mM L-Ala-*p*-nitroanilide to determine the optimal temperature. For the stability analysis, eAPN was incubated at 37 °C and 4 °C for up to 6 days and sampled at intervals. Residual activity was determined to evaluate its stability at 37 °C and 4 °C.

To ascertain the melting temperature (*T*_m_) of eAPN, purified enzyme was diluted to a final concentration of 20 μM and mixed with SYPRO^®^ Orange dye at a final concentration of 5×. This mixture was then aliquoted into a 96-well plate, with each well receiving a total volume of 10 μL. An optically transparent adhesive sheet was applied to seal the wells, followed by centrifugation at 800× *g* for 2 min. Finally, the analysis was conducted using the Applied Biosystems Quant Studio 6 real-time PCR system (Bio-Rad, Hercules, CA, USA). The reaction was initiated at 25 °C and gradually heated at a rate of 1 °C/min until it reached a final temperature of 95 °C. Fluorescence intensity was monitored throughout the reaction to collect data. The protein melting temperature was determined by fitting the dataset to a Boltzmann sigmoidal curve using GraphPad Prism 10, employing the equation: Y = bottom + (top − bottom)/(1 + exp ((Tm − X)/slope)).

### 2.7. Substrate Specificity of eAPN Toward Aminopeptidase Substrates

Previous investigations have unequivocally established the catalytic proficiency of eAPN in hydrolyzing L-Ala-*p*-nitroanilide [24,25]. To further elucidate its substrate specificity, six peptidase substrates including L-Ala-*p*-nitroanilide, L-Arg-*p*-nitroanilide, L-Leu-*p*-nitroanilide, L-Gly-*p*-nitroanilide, L-Met-*p*-nitroanilide, and L-Pro-*p*-nitroanilide, were selected for analysis. These substrates were tested in a concentration range of 50 μM to 4 mM for kinetic analysis. Based on enzyme activity levels for specific substrates, the concentration ranges of eAPN (2.5 nM–200 nM) and His-eAPN (25 nM–2 µM) were employed accordingly. Reactions were performed at 37 °C in 50 mM phosphate buffer (pH 7.5). The method for determining enzyme activity remains the same as previously described. The kinetic parameters, including *k*_cat_ and *K*_M_, were estimated by performing the Michaelis–Menten kinetic analysis using Prism 10 (GraphPad Software Inc., Boston, MA, USA).

### 2.8. Molecular Modelling

The interactions of heroin and 6-MAM with eAPN were modeled using a protocol similar to our previous research [22]. Initially, the enzyme’s coordinates were sourced from the X-ray crystallography data available in the Protein Data Bank (PDB ID 2ZXG), which shows eAPN in complex with the aminophosphinic inhibitor PL250. This molecule mimics the transition state. Heroin and 6-MAM were manually constructed by modifying the structure of PL250. CHARMM HBUILD module was used to complete the protein model to add hydrogen atoms [26]. The protonation states of the amino acid residues were set according to their potential hydrogen bonding at pH 7.4, as determined by CHARMM-GUI. The zinc atom (Zn) was the central focus of our model. The solvent environment was simulated by placing a water sphere with a 22 Å radius around the complex. Both the solvent and any water molecules present in the crystal structure were modeled using an enhanced TIP3P water model [27,28]. For the enzyme minimization using a QM/MM approach, the substrates were treated quantum mechanically, while the rest of the system was handled classically. The SCC-DFTB method, integrated within CHARMM, was applied to the QM atoms [29], and the CHARMM36m force field was employed for the MM atoms [30].

### 2.9. Catalytic Activity of eAPN Toward 6-MAM and Heroin

The enzymatic activities of eAPN toward heroin and 6-MAM were investigated under a well-defined assay condition. Specifically, 50 μL of purified enzyme was combined with 50 μL of either heroin or 6-MAM in 50 mM phosphate buffer solution (pH 7.5). For kinetic analysis, 20 μM enzyme was used, and the substrates were tested in a concentration range of 50 μM to 5 mM. Reactions were conducted at 37 °C for 6 h (heroin) or 24 h (6-MAM) to ensure that substrate depletion did not exceed 30%. The reactions were terminated, and protein precipitation was achieved by the addition of 50 μL of 0.5 M hydrochloric acid and 150 μL of 20% acetonitrile, followed by a 10 min centrifugation at 12,000 rpm. The supernatant was subjected for product quantification using the HPLC method.

RP-HPLC analysis using Thermo Ultimate 3000 HPLC-UV detection system (Thermo Fisher Scientific, Waltham, MA, USA) was performed on a Welch xtimate^®^ C18 column (250 × 4.6 mm, 5 μm). Gradient elution with 1 mL/min flow rate was employed using 0.1% trifluoroacetic acid (TFA) as mobile phase A and acetonitrile as mobile phase B, and the elution program was set as follows: 0–2 min, 10% B; 2–20 min, 10–25% B; 20–24 min, 25% B; 24–26 min 25–10% B; 26–28 min, 10% B. The remaining substrate and the formed products in the reaction mixture were monitored at 210 nm. The peaks of morphine, 6-MAM, and heroin appeared at 5.50 min, 13.75 min, and 23.60 min, respectively. The quantification of analytes in the reaction mixture was achieved using a standard curve prepared with authentic standard compounds. Kinetic parameters were analyzed using the method previously outlined.

## 3. Results

### 3.1. Sequence Analysis

**Figure 2:**
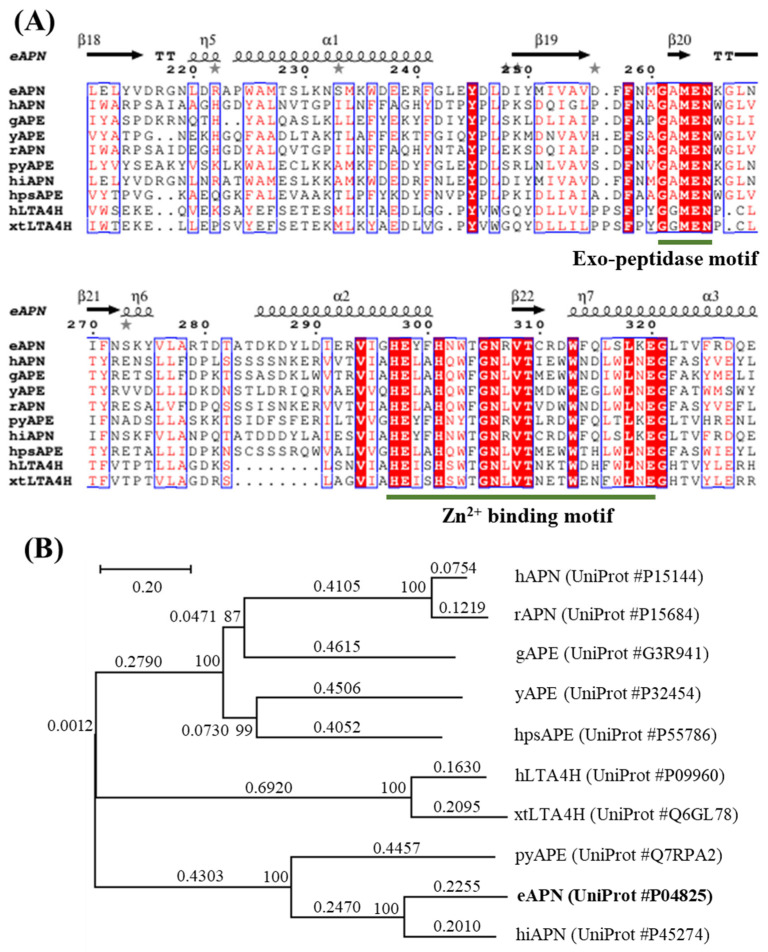
Sequence alignment and phylogenetic analyses of eAPN with other M1 family enzymes. (**A**) The sequence alignment of ten enzymes, with the key features including the site of zinc binding motif and exopeptidase motif underlined for clarity. (**B**) The phylogenetic tree for eAPN and its structural homologs was constructed using the neighbor-joining method with MEGA7 software. The tree is drawn to scale, with branch lengths representing evolutionary distances in units of amino acid substitutions per site. The scale bar indicates 0.20 substitution per nucleotide position. Numbers at nodes are bootstrap percentages derived from 1000 resampled datasets.

The eAPN cDNA consists of 2610 nucleotides and encodes a protein composed of 870 amino acids, with an estimated molecular weight of 98.9 kDa. To investigate the sequence similarities within the M1 family of proteins, the following nine additional members were selected for comparison: human aminopeptidase N (hAPN), human leukotriene A4 hydrolase (hLTA4H), human puromycin-sensitive aminopeptidase (hpsAPE), gorilla aminopeptidase (gAPE), rat aminopeptidase N (rAPN), plasmodium yoelii aminopeptidase (pyAPE), xenopus tropicalis leukotriene A4 hydrolase (xtLTA4H), haemophilus influenzae aminopeptidase N (hiAPN), and yeast aminopeptidase 2 (yAPE). Using the BLASTp tool, the amino acid sequences of these nine proteins with that of eAPN were aligned, and their amino acid sequence similarity ranged from 22% to 61%. The specific similarities are as follows: xtLTA4H 22.57%, hLTA4H 24.40%, gAPE 25.06%, rAPN 25.63%, yAPE 26.09%, hpsAPE 26.22%, hAPN 27.01%, pyAPE 34.66%, and hiAPN 60.67%. The alignment of amino acid sequences reveals that these proteins of the M1 family possess a distinctive zinc-binding motif (HEXXH-(X18)-E), as well as a conserved exopeptidase motif (GXMEN) in their active sites (Figure 2A). The phylogenetic analysis of these ten sequences places them into three distinct branches (Figure 2B). eAPN, pyAPE, and hiAPN form one branch; xtLTA4H and hLTA4H form the second branch; hAPN, rAPN, gAPE, yAPE, and hpsAPE form the third branch. This analysis confirms that eAPN and hiAPN have the highest sequence similarity.

### 3.2. Cloning, Expression, and Purification of Recombinant eAPN

**Figure 3:**
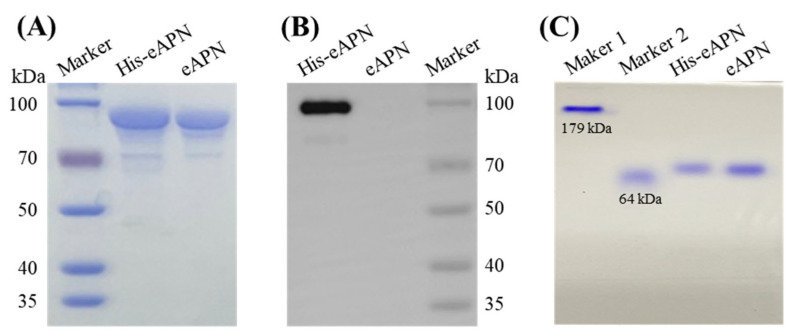
Protein gel analysis of purified eAPN and His-eAPN. (**A**) SDS-PAGE. (**B**) Western blot using anti-His antibody. (**C**) Native PAGE marker 1 and marker 2 are in-house proteins, whose theoretical MW are 179 kDa and 64 kDa, respectively. Original images can be found in Supplementary Materials.

To prepare recombinant eAPN protein for biochemical and functional characterization, the cDNA encoding eAPN was cloned into the pET28a vector. Its expression was induced with IPTG in *E. coli* BL21 cells, and its purification was carried out using Ni-NTA chromatography and thrombin digestion. In this procedure, His-eAPN is the originally expressed format, and 30 mM imidazole was applied for His-eAPN elution in affinity chromatography (Figure S1). Although thrombin for tag cleavage is confirmed not to interfere with the enzyme activity assay using L-Ala-*p*-nitroanilide as the substrate (Figure S2), thrombin was separated with tag-free eAPN by dialyzing with Amicon Ultra-50 kD centrifugal filters. The experimental results reveal that eAPN showed a significant level of expression, with a yield of ~90 mg/L. The resulting eAPN protein exhibited a purity exceeding 90% and a molecular weight close to 100 kDa (Figure 3A). The SDS-PAGE analysis shows no notable shift in the protein band position upon His-tag removal; however, the Western blotting using anti-His antibodies confirms the complete excision of the His-tag (Figure 3B). The Native PAGE analysis demonstrates that the His-tag had no impact on the aggregation state of eAPN (Figure 3C).

### 3.3. Effect of pH, Thiol Reducer, Metal Ions

**Figure 4:**
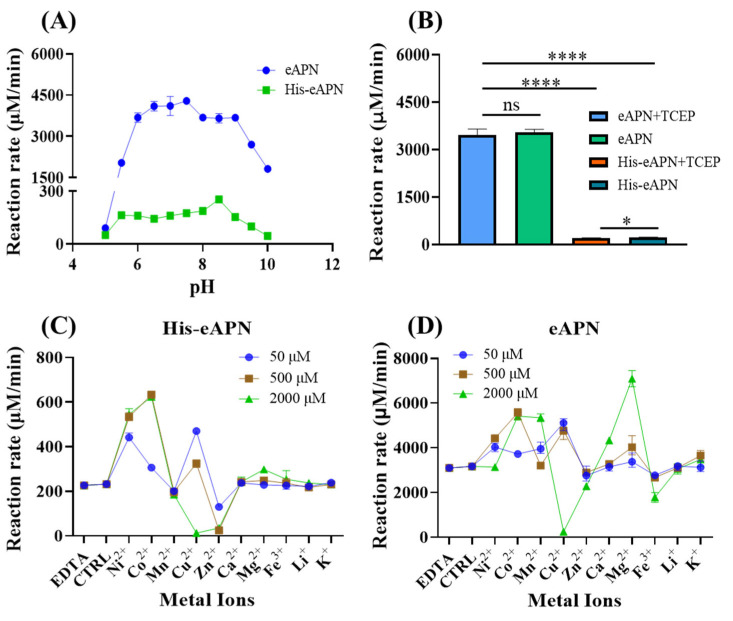
The effects of pH, thiol reducer, and metal ions on the activity of eAPN and His-eAPN. (**A**) The impact of pH in the range of 5.0–10.0. (**B**) The effect of 500 μM TCEP at pH 7.5. (**C**,**D**) The effect of various metal ions at different concentrations on His-eAPN (**C**) and eAPN (**D**) at pH 7.5. Reaction rate refers to the normalized reaction velocity presented in μM/min per μM of enzyme. The error bar indicates the standard deviation of triplicate data. Significance: ns (*p* > 0.05), * (*p* ≤ 0.05), **** (*p* ≤ 0.0001).

To evaluate the effect of pH on the activity of eAPN, assessment was conducted across a pH range of 5.0 to 10.0. The results indicate that eAPN retained over 85% of its enzyme activity across the pH range from 6.0 to 9.0 (Figure 4A). Notably, the optimal activity of eAPN was observed at pH 7.5; whereas, His-eAPN exhibited peak activity at pH 8.5. According to the optimal pH of eAPN, which is almost equal to the physiological condition, the following enzymatic assays were all performed at pH 7.5. Furthermore, the impact of TCEP, a potent reducing agent capable to break disulfide bonds, on the enzyme activity of eAPN was also investigated. In total, 500 μM TCEP was introduced to the experimental group, and subsequent activity analysis reveals that the effect of TCEP on eAPN was insignificant (Figure 4B), suggesting TCEP does not decrease the enzyme’s activity, and it may be used for long-term storage. In addition, the results of Figure 4 reveal the N-terminal His-tag significantly reduced eAPN’s aminopeptidase activity, suggesting the harmful effects of His-tag to eAPN’s catalytic activity against L-Ala-*p*-nitroanilide and the necessity of tag removal to reflect eAPN’s native property and function.

eAPN was recognized as a zinc-dependent aminopeptidase with the zinc ion presenting in the active site at full occupancy [31,32]; however, there remains an ongoing debate concerning the necessity of metal ions for eAPN activity [25,32,33]. Therefore, the impacts of various metal ions at different concentrations on eAPN enzyme activity were evaluated in this study. Compared to the control group, a 10 mM EDTA addition did not affect the enzymatic activity of both eAPN and His-eAPN. As illustrated in Figure 4D, most metal ions demonstrated activation effects to eAPN, such Co^2+^, Ni^2+^, Cu^2+^, and Mg^2+^. Cu^2+^ functioned as an activator at lower concentrations but underwent a transition into an inhibitor beyond 500 μM, ultimately causing near-complete inactivation at 2 mM. Zn^2+^ presented a slight inhibitory effect on eAPN, which was further confirmed in a dose-response study and EDTA restoration test (Figure S3). In the presence of N-terminal His-tag, the effects of metal ions on eAPN’s aminopeptidase activity are roughly consistent. At a concentration of 500 μM, the order of metal ion’s activation effects on His-eAPN is discernible as Co^2+^ > Ni^2+^ > Cu^2+^ > Ca^2+^, Mg^2+^, Fe^3+^, Li^+^, K^+^ > Zn^2+^ (Figure 4C). Notably, Co^2+^ and Ni^2+^ demonstrated the most pronounced stimulatory effects on His-eAPN, and Zn^2+^ demonstrated stronger inhibition on His-eAPN than eAPN. Both His-eAPN and eAPN were almost completely inhibited by high concentrations of Cu²⁺, likely due to the metal’s inherent toxicity to proteins. Although eAPN was known to be zinc-dependent [32,34], there is literature reporting that Zn²⁺ has an inhibitory effect on the enzymatic activity of eAPN [25,33]. According to above results, it appears Zn^2+^ does not work as an activator of eAPN in catalyzing L-Ala-*p*-nitroanilide hydrolysis.

### 3.4. Effect of Temperature and Thermostability of eAPN

**Figure 5:**
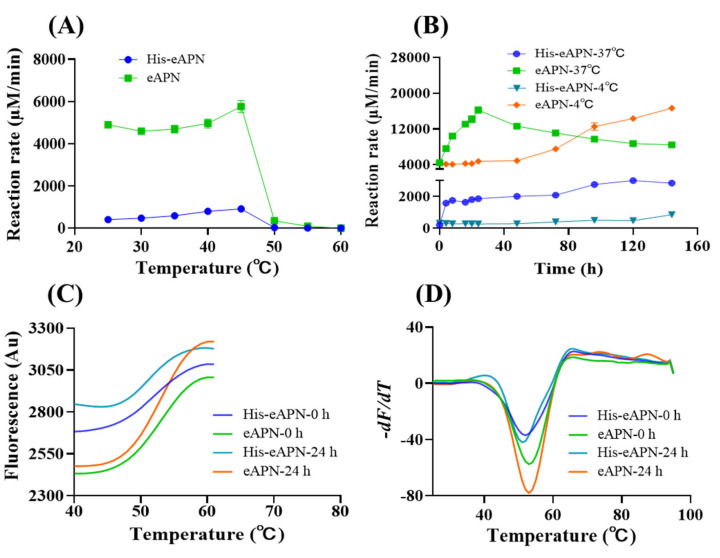
The thermal stability of eAPN and His-eAPN. (**A**) The effect of temperature on enzyme activity at pH 7.5. (**B**) Residual activity of enzymes following incubation at 37 °C and 4 °C for various durations at pH 7.5. Reaction rate refers to the normalized reaction velocity presented in μM/min per μM of enzyme. (**C**) Automated processing of thermal denaturation curves, with the dataset associated with post-peak quenching truncated. *T*_m_ is represented as the midpoint of the curve’s transition. (**D**) Determination of *T*_m_. *T*_m_ is identified as the lowest point of the curve.

To evaluate the effect of temperature on eAPN activity, the enzyme was incubated at temperatures ranging from 25 to 60 °C for 30 min, followed by the determination of aminopeptidase activity. The results indicate that both eAPN and His-eAPN exhibited peak activity for L-Ala-*p*-nitroanilide at 45 °C (Figure 5A). However, as the temperature increased, the activity of both enzymes decreased dramatically beyond 45 °C, leading to nearly complete loss of activity by 60 °C.

To gain a deeper insight into the stability of eAPN at both 4 °C and 37 °C, samples of eAPN and His-eAPN were incubated for a span of 0 to 6 days to evaluate their residual enzyme activity toward L-Ala-*p*-nitroanilide. The experimental data reveals a similar activation trend in both enzymes at 37 °C. Specifically, eAPN activity surged following incubation and peaked at 24 h (Figure 5B). His-eAPN activity also surged following incubation and slightly increased from 24 h to 6 days at 37 °C. Even after 6 days of incubation at 37 °C, eAPN activity exceeded its initial level by approximately 1.9 times, while His-eAPN activity maintained a level that was nearly 13.5 times its initial value. When incubated at 4 °C, the activity levels of both eAPN and His-eAPN remained stable before 48 h. Their activity levels began to rise steadily from 48 h until the endpoint. These results suggest incubation temperature is crucial for eAPN’s activation. This study not only highlights eAPN’s remarkable thermal stability but also provides insights into its intriguing heat activation mechanism. During incubation at 37 °C, no oligomerization change in eAPN was observed (Figure S4). However, a conformational change might occur when the temperature shifts from 4 °C to 37 °C. The detailed conformational change underlying this phenomenon may be elucidated through long-term molecular dynamics (MD) simulations conducted at these specific temperatures. Such studies are suggested as a direction for future research, which could potentially explain the observed heat activation of eAPN.

To further characterize the thermal stability of eAPN, a quantitative assessment of its melting temperature (*T*_m_) was conducted. Given the notable enhancement in eAPN activity following 24 h at 37 °C, the effect of varying storage durations at this temperature on the *T*_m_ value of eAPN was investigated. The experimental results indicate that His-eAPN and eAPN exhibit a consistent *T*_m_ of 51–52 °C, and 53 °C, respectively, regardless of whether they were pre-incubated at 37 °C for 24 h or not (Figure 5C,D). This finding further highlights the inherent thermal stability of eAPN and also demonstrates that the presence of the His tag does not significantly alter the *T*_m_ value of eAPN, despite its inhibitory effect on the enzyme’s activity.

### 3.5. Substrate Specificity of eAPN Toward Peptidase Substrates

**Figure 6:**
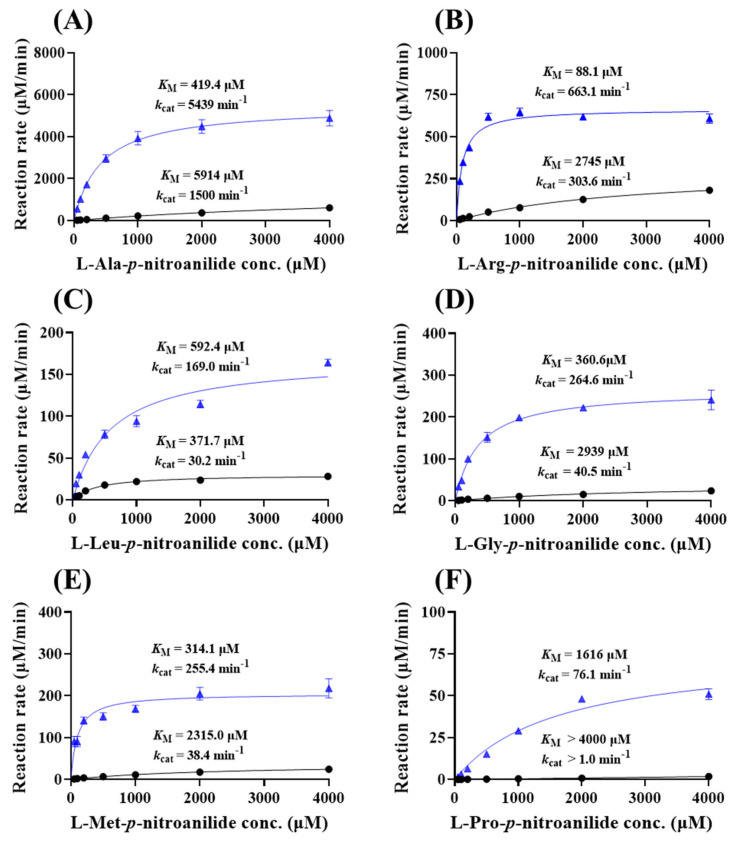
Michaelis–Menten kinetics of eAPN and His-eAPN for aminopeptidase substrates at 37 °C, pH 7.5. (**A**) L-Ala-*p*-nitroanilide; (**B**) L-Arg-*p*-nitroanilide; (**C**) L-Leu-*p*-nitroanilide; (**D**) L-Gly-*p*-nitroanilide; (**E**) L-Met-*p*-nitroanilide; (**F**) L-Pro-*p*-nitroanilide. The kinetic data of eAPN (blue triangles) and His-eAPN (black circles) were presented in each figure. The initial reaction velocity is represented in μM/min per μM of enzyme, with error bars indicating the standard deviation of three replicates.

To delve into the substrate specificity of eAPN, a comprehensive kinetic analysis using six aminopeptidase substrates—L-Ala-*p*-nitroanilide, L-Arg-*p*-nitroanilide, L-Leu-*p*-nitroanilide, L-Gly-*p*-nitroanilide, L-Met-*p*-nitroanilide and L-Pro-*p*-nitroanilide—was conducted. As shown in Figure 6 and Table 1, eAPN exhibited the highest catalytic efficiency (*k*_cat_/*K*_M_) when catalyzing L-Ala-*p*-nitroanilide, followed by L-Arg-*p*-nitroanilide. In contrast, it demonstrated the lowest catalytic efficiency toward L-Pro-*p*-nitroanilide. A similar substrate preference was observed in both eAPN and His-eAPN. This establishes a clear preference hierarchy of eAPN in substrate hydrolysis, as follows: Ala > Arg >> Met, Gly > Leu > Pro. The extensive substrate specificity profile not only underscores the versatility of eAPN aminopeptidase activity but also suggests a preference for amino acid residues that are either small or basic.

In addition, eAPN demonstrates a lower Michaelis–Menten constant (*K*_M_), i.e., higher binding affinity and higher catalytic constant (*k*_cat_), for catalyzing these six substrates than His-eAPN. Consequently, the catalytic efficiency (*k*_cat_/*K*_M_) of eAPN is significantly enhanced compared to His-eAPN. To investigate the substrate binding effect induced by the His-tag to eAPN, AlphaFold3 was utilized to conduct homology modeling and performed docking studies with L-Ala-*p*-nitroanilide. As depicted in Figure S5, the addition of the N-terminal His-tag does not significantly alter the overall structure, with an RMSD of 0.105 Å. However, the substrate binding characteristics differ between the two variants. In the absence of the N-terminal His-tag, eAPN exhibits stronger hydrophobic interactions between the aromatic rings of His297 and the substrate compared to His-eAPN. This enhanced interaction in the eAPN complex likely provides greater stabilization energy in both the reaction state and the transition state, thereby contributing to improved catalytic efficiency.

### 3.6. Catalytic Activity of eAPN Toward 6-MAM and Heroin

**Figure 7:**
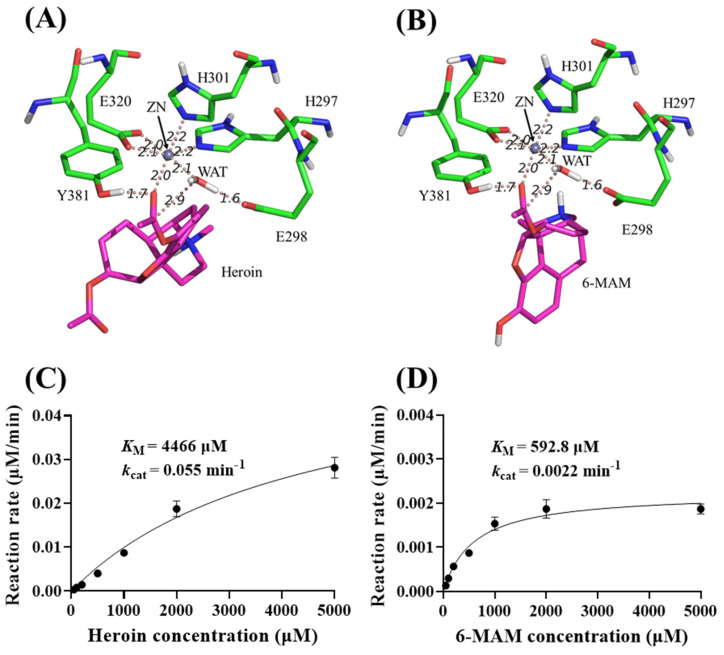
Molecular modeling and kinetic data of eAPN-catalyzed heroin and 6-MAM hydrolysis. (**A**,**B**) The energy-minimized ES structures for heroin (**A**) and 6-MAM (**B**) interacting with eAPN. (**C**,**D**) Michaelis–Menten kinetic data of eAPN toward heroin (**C**) and 6-MAM (**D**) at 37 °C, pH 7.5. Reaction rates are presented as mean ± standard deviation in μM/min per μM enzyme.

Insights from molecular modeling. A computational search suggests eAPN may be capable of catalyzing the hydrolysis of 6-MAM and heroin. Our research has shed light on the reactant structures of eAPN when complexed with these substrates. Figure 7A,B illustrates the optimized reactant complexes, which were derived from QM/MM-based minimizations. These complexes highlight key enzyme–substrate interactions. Notably, the acetyl groups of heroin and 6-MAM interact with the oxyanion hole, which is formed by the sidechain hydroxyl group of Tyr381 and the positively charged Zn ion. Additionally, the catalytic residues exhibit distinctive properties. A nucleophilic water molecule is hydrogen-bonded to the general base residue Glu298, with the nucleophilic distance between the water’s oxygen atom and the acetyl carbon atom of the substrates being approximately 2.9 Å. These structural insights suggest that the reactions are highly likely to proceed.

Capability of eAPN to hydrolyze heroin and 6-MAM. To evaluate the catalytic activity of eAPN toward heroin and 6-MAM, an HPLC-UV method was established to quantify heroin and its metabolites. The gradient elution using acetonitrile and 0.1% TFA as mobile phases was performed for better separation. Under this method, heroin, 6-MAM, and morphine exhibit a retention time of 23.60 min, 13.75 min, and 5.50 min, respectively. Compared to the enzyme-free group, which serves as a control for the spontaneous hydrolysis of heroin and 6-MAM, the experimental group with eAPN under the same conditions exhibited higher peak of reaction products (Figure S6). This observation strongly suggests that eAPN is capable of catalyzing the hydrolysis of both heroin and 6-MAM.

To further characterize the catalytic efficiency of eAPN toward heroin and 6-MAM, a detailed kinetic analysis was carried out to determine their kinetic parameters of these substrates. The results in Figure 7C,D reveal that eAPN catalyzed the hydrolysis of both substrates, exhibiting *k*_cat_ = 0.055 min^−1^ and *K*_M_ = 4466 μM for heroin and *k*_cat_ = 0.0022 min^−1^ and *K*_M_ = 592.8 μM for 6-MAM. Notably, eAPN demonstrated a lower *K*_M_ value for 6-MAM than heroin, suggesting a stronger binding affinity for 6-MAM. Despite its relatively low catalytic activity, the ability of eAPN to hydrolyze both heroin and 6-MAM highlights its versatility as a biocatalyst for esters and potential of reengineering for degradation applications.

## 4. Discussion

6-MAM is recognized as the primary active metabolite that reaches the brain, making it a crucial factor in heroin-induced physiological effects and lethal toxicity. Currently, enzyme therapy for addiction, particularly cocaine abuse, has attracted significant attention because of its proven efficacy in clinical trials [15,16,17]. Utilizing a computational approach, an aminopeptidase derived from *E. coli* was identified with the potential to catalyze the hydrolysis of 6-MAM. This study reveals for the first time that eAPN can indeed catalyze heroin and 6-MAM hydrolysis. Its biochemical and functional properties were extensively investigated in order to gain a better understanding of the enzyme itself and its potential for reengineering as a detoxifying agent for 6-MAM.

eAPN is a member of the M1 family of peptidases, which includes a wide range of metalloproteases categorized under the gluzincin classification [32]. A distinctive feature of M1 aminopeptidases is the presence of a conserved zinc-binding motif (HEXXH-(X18)-E), along with an exopeptidase motif (GXMEN), both located in their active sites [24,35]. Although each member of the M1 family performs distinct physiological roles, they all function uniformly as aminopeptidases [23,33,36]. eAPN plays a crucial role in the final stages of peptide digestion and participates in the metabolic decomposition of bioactive peptides [24]. Previous studies have demonstrated that eAPN predominantly catalyzes the hydrolysis of substrates with alanine at the N-terminus, accounting for approximately 99% of its enzyme activity [37,38]. However, it has been suggested that eAPN shows increased specificity for alkaline amino acid residues, such as arginine and lysine [33]. Kinetic data derived from this study confirms that eAPN does not function exclusively as an alanine aminopeptidase. While it shows the highest catalytic efficiency toward L-Ala-*p*-nitroaniline, L-Arg-*p*-nitroaniline is also a highly preferred substrate. The preferential order of activity is as follows: Ala > Arg >> Met, Gly > Leu > Pro, suggesting a broad substrate scope of eAPN in protein degradation. These findings also highlight the substrate specificity of eAPN, with the highest specificity for small hydrophobic residues, such as alanine, followed by the basic residue arginine. The outcome may be attributable to the restriction imposed by the side chain of Met260 on the S1 subsite of eAPN. This confinement results in a subsite cavity that is ideally suited for accommodating smaller residues, notably alanine [31]. Intriguingly, eAPN also demonstrates the capacity to cleave L-Pro-*p*-nitroaniline, a proline-containing substrate that is typically resistant to degradation by most enzymes. This feature further underscores the function versatility and potential of eAPN [24].

In search of potential 6-MAM hydrolase, the simulated eAPN structures in complex with 6-MAM or heroin indicate the potential of eAPN catalyzing 6-MAM or heroin. Experimental results confirm that eAPN exhibits catalytic activity toward 6-MAM or heroin. Importantly, kinetic parameters derived from this study indicate that eAPN has a significantly higher binding affinity for 6-MAM compared to heroin, as evidenced by a 5-fold difference in their *K*_M_ values. Although the interactions between the key residues in the enzyme active site and heroin or 6-MAM are similar, the substrates differ at the 3′ position sidechain; heroin has an acetyl group, while 6-MAM has a hydroxyl group. As shown in Figure S7, the acetyl oxygen of heroin forms a hydrogen bond network with a water molecule and R825 sidechain, while the hydroxyl group of 6-MAM forms a hydrogen bond network with a water molecule and R825 sidechain. Considering that the hydroxyl group is more polar than the acetyl group, the hydrogen bond network in 6-MAM complex may contribute more binding energy than that of heroin complex. Although eAPN shows a lower catalytic efficiency than known endogenous 6-MAM hydrolases including cholinesterases and carboxylesterases [10,22,39], its high recombinant yield and satisfactory biochemical performance, such as thermostability and optimal pH of 7.5, provides a strong foundation for future development. With our enzyme design method [40,41] potentially further enhancing its catalytic activity, eAPN holds great potential for heroin detoxification. This work elucidates the biochemical properties of eAPN and expands its catalytic functions, which lay the groundwork for a deep understanding of eAPN and its further reengineering for enhanced performance and applications. Additionally, the N-terminal His-tag significantly interferes with eAPN’s catalytic activity and biochemical properties. The comparison of eAPN’s properties in its apo and His-tagged forms suggests the need for caution when including tags in protein for its functional study.

## 5. Conclusions

To summarize, in the quest of a search for a novel 6-MAM hydrolase, this study not only discovered the new catalytic function of eAPN, an *E. coli* aminopeptidase as a 6-MAM hydrolase, but also comprehensively elucidates its biochemical properties. Its substrate selectivity characterization toward peptidase substrates identifies it not merely as an alanine aminopeptidase, thereby solidifying the existing knowledge regarding its physiological function in peptide digestion. The identification of eAPN’s novel role in hydrolyzing 6-MAM has shed light on the multifaceted landscape of eAPN. It is cost-effective in production and has favorable thermostability, providing a foundation for revealing its untapped potential in the detoxification of heroin and 6-MAM. Further investigation is warranted to further optimize its catalytic function through rational enzyme design strategies.

## Figures and Tables

**Table 1:** Kinetic parameters determined for the hydrolysis of aminopeptidase substrates catalyzed by eAPN and His-eAPN at 37 °C.

Enzyme	Substrate	*k*_cat_ (min^−1^)	*K*_M_ (μM)	*k*_cat_/*K*_M_ (M^−1^min^−1^)
His-eAPN	L-Ala-*p*-nitroanilide	1500.0	5914.0	0.25 × 10^6^
	L-Arg-*p*-nitroanilide	303.6	2745.0	0.11 × 10^6^
	L-Met-*p*-nitroanilide	38.4	2315.0	0.02 × 10^6^
	L-Gly-*p*-nitroanilide	40.5	2939.0	0.01 × 10^6^
	L-Leu-*p*-nitroanilide	30.2	371.7	0.08 × 10^6^
	L-Pro-*p*-nitroanilide	>1.0	>4000	/
eAPN	L-Ala-*p*-nitroanilide	5439.0	419.4	12.97 × 10^6^
	L-Arg-*p*-nitroanilide	663.1	88.1	7.53 × 10^6^
	L-Met-*p*-nitroanilide	255.4	314.1	0.81 × 10^6^
	L-Gly-*p*-nitroanilide	264.6	360.6	0.73 × 10^6^
	L-Leu-*p*-nitroanilide	169.0	592.4	0.29 × 10^6^
	L-Pro-*p*-nitroanilide	76.1	1616.0	0.05 × 10^6^

## Data Availability

The original contributions presented in this study are included in the article/Supplementary Materials. Further inquiries can be directed to the corresponding author.

## Supplementary Materials

The following supporting information can be downloaded at https://www.mdpi.com/article/10.3390/biom15060822/s1, Figure S1: SDS–PAGE analysis of recombinant His-eAPN purification process. Figure S2: The effects of thrombin on L-Ala-*p*-nitroanilide hydrolysis. Figure S3: The effect of Zinc ions on eAPN and His-eAPN. Figure S4: Native-PAGE analysis of purified eAPN and His-eAPN incubated at 37℃. Figure S5: Superimposed Alphafold3 docking results for His-eAPN and eAPN. Figure S6: HPLC separation and UV detection of heroin, 6-MAM, and morphine. Figure S7: Hydrogen bond network in the energy-minimized ES structures for heroin and 6-MAM interacting with eAPN. Original images of Figure 3 can be found in supplementary materials.
