# Supplementary material for: Biochemical and Functional Characterization of E. coli Aminopeptidase N: A New Role as a 6-Monoacetylmorphine Hydrolase
Source: Biomolecules. 2025 Jun 5;15(6):822. doi: 10.3390/biom15060822 (PMC12190285; doi:10.3390/biom15060822)
Supplement: Supplementary file 1 [file biomolecules-15-00822-s001.zip › biomolecules-3632269-original-images.pdf]

Original gel images for Figure 3 in the manuscript

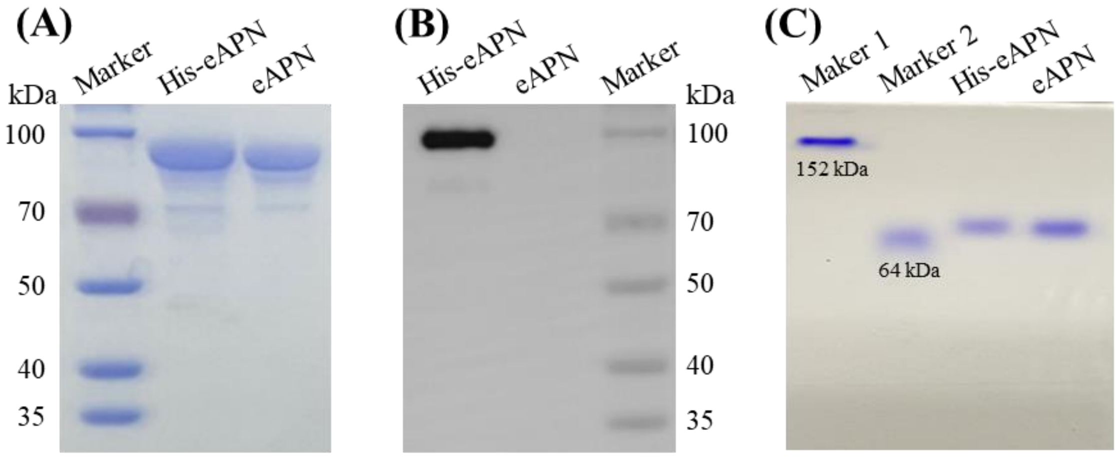

Figure 3. Protein gel analysis of purified eAPN and His-eAPN. (A) SDS-PAGE. (B) Western Blot using anti-His antibody. (C) Native-PAGE, marker 1 and marker 2 are in house proteins, whose theoretical MW are 152 kDa and 64 kDa, respectively.

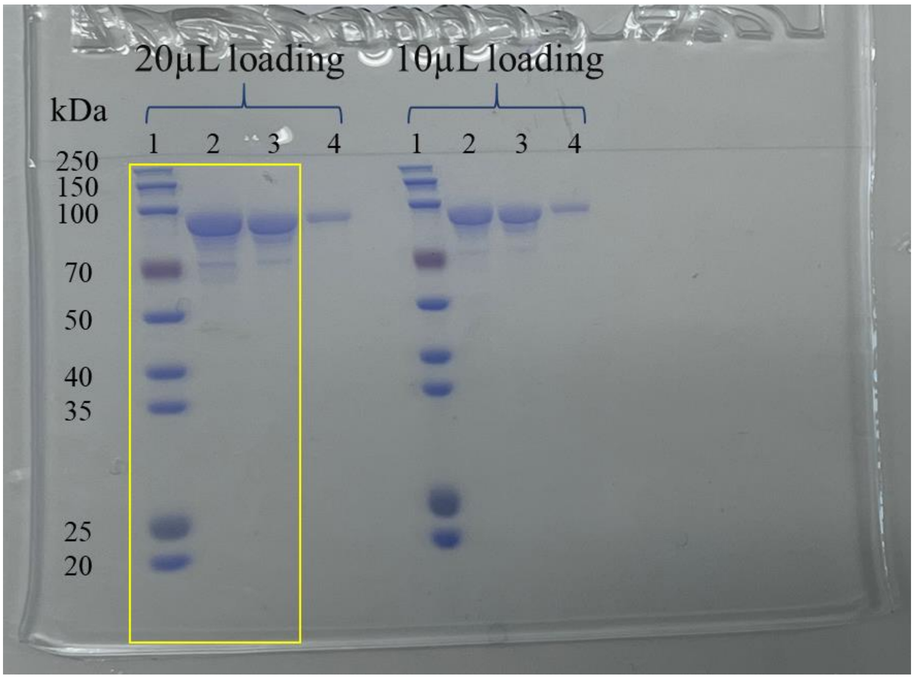

Original Figure 3A. SDS-PAGE of eAPN and His-eAPN:

1. Marker; 2. His-eAPN (2/1 μg); 3. eAPN (2/1 μg); 4. eAPN (0.5/0.25 μg)

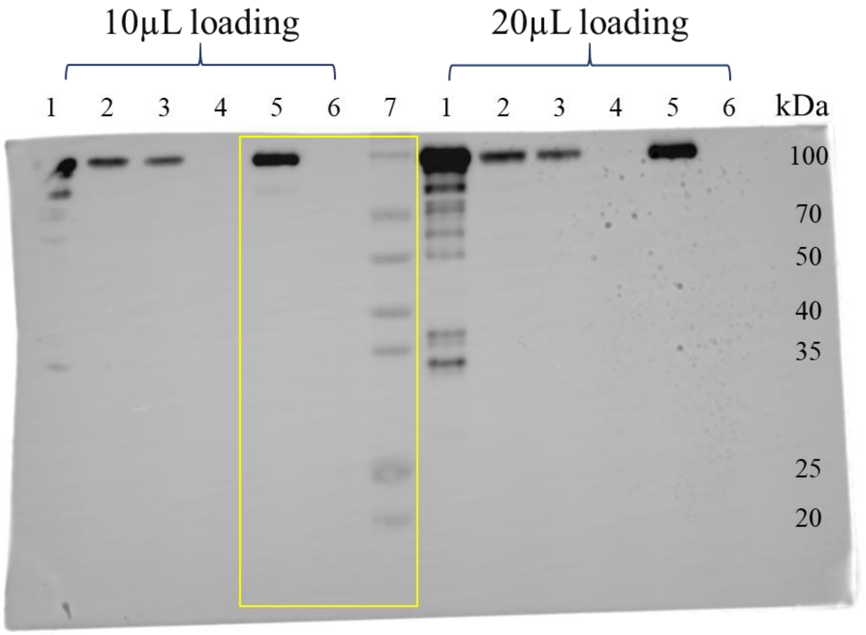

Original Figure 3B. Western Blot of eAPN and His-eAPN using anti-His antibody:

1. His-eAPN crude sample (0.5/1 μg)
2. His-eAPN (before thrombin treatment, 0.1/0.2 μg)
3. His-eAPN (2hr treatment with thrombin, 0.1/0.2 μg)
4. eAPN (16hr treatment with thrombin, 0.1/0.2 μg)
5. His-eAPN (before thrombin treatment, 0.2/0.4 μg)
6. eAPN (16hr treatment with thrombin, 0.2/0.4 μg)
7. Marker

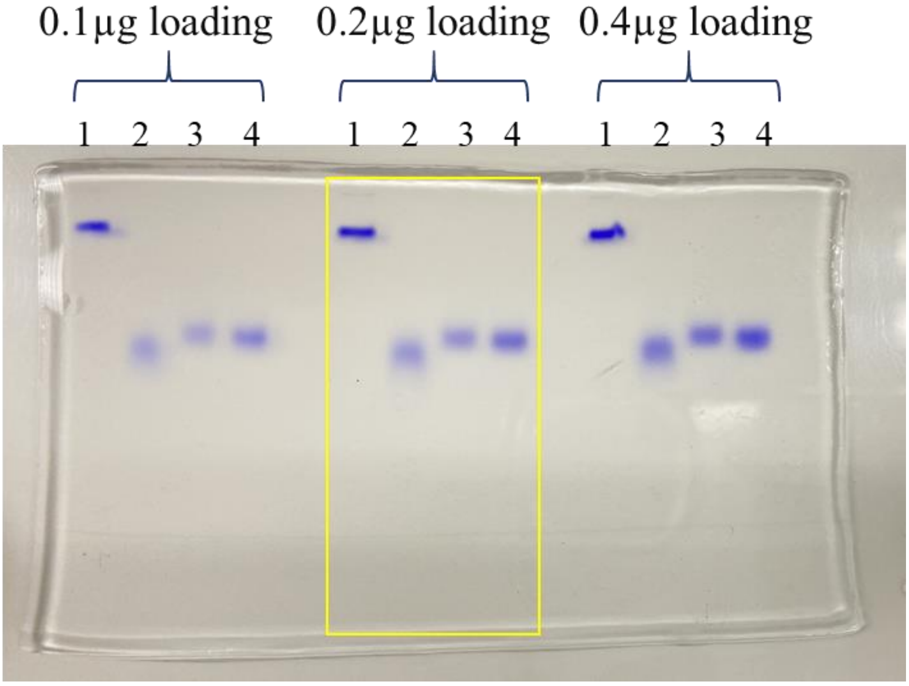

Original Figure 3C. Native-PAGE of eAPN and His-eAPN:

1. Marker 1—CES2-Fc (in house, *dimer*, MW_T=152.4 kDa)
2. Marker 2—CES2 (in house, *monomer*, MW_T=63.6 kDa)
3. His-eAPN (*monomer*, MW_T=100.9 kDa)
4. eAPN (*monomer*, MW_T=98.9 kDa)
