# Supplementary material for: Biochemical and Functional Characterization of E. coli Aminopeptidase N: A New Role as a 6-Monoacetylmorphine Hydrolase
Source: Biomolecules. 2025 Jun 5;15(6):822. doi: 10.3390/biom15060822 (PMC12190285; doi:10.3390/biom15060822)
Supplement: Supplementary file 1 [file biomolecules-15-00822-s001.zip › Biomolecules-3632269-Supplementary.pdf]

Supplementary Information for

Biochemical and functional characterization of E. coli aminopeptidase N: A new role as a 6-monoacetylmorphine hydrolase (Manuscript#: Biomolecules-3632269)

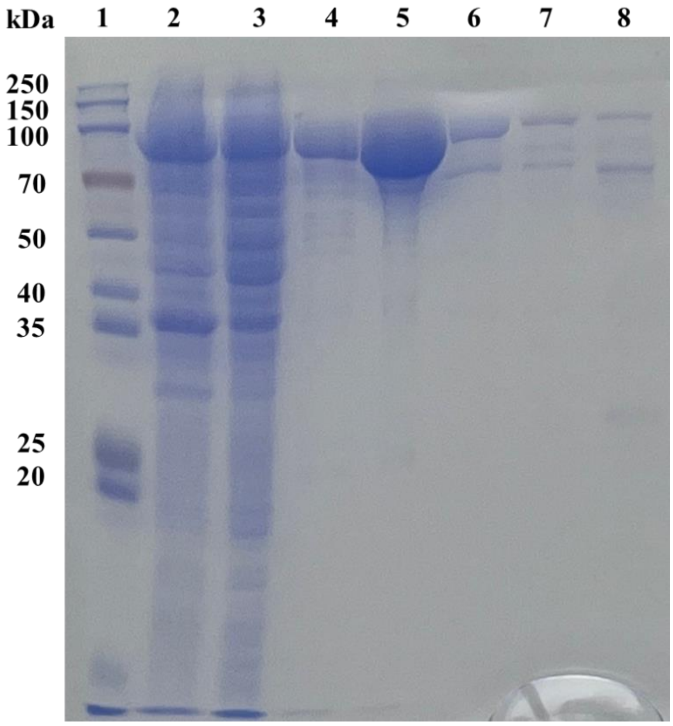

Figure S1. SDS–PAGE analysis of recombinant His-eAPN purification process. The supernatant from cell lysis, flow-through after binding and elution fractions using 10 mM, 30 mM, 50 mM, 100 mM, and 300 mM imidazole were collected for gel analysis. Lane 1: Marker; Lane 2: Cell lysate; Lane 3: Flow-through; Lane 4-8: Elution fractions using 10 mM, 30 mM, 50 mM, 100 mM, and 300 mM imidazole, respectively.

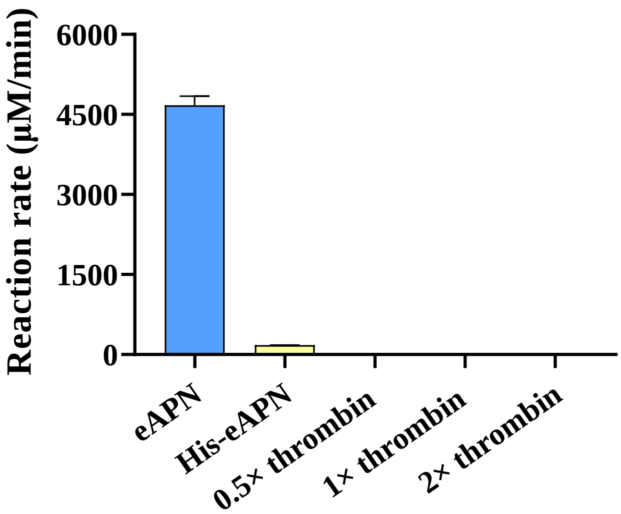

Figure S2. The effects of thrombin on L-Ala-*p*-nitroanilide hydrolysis. eAPN and His-eAPN serve as the control. 1×thrombin refers to the concentration of thrombin in His-tag cleavage system; 2×thrombin and 0.5×thrombin correspond to twice or half the concentration of thrombin in His-tag cleavage system.

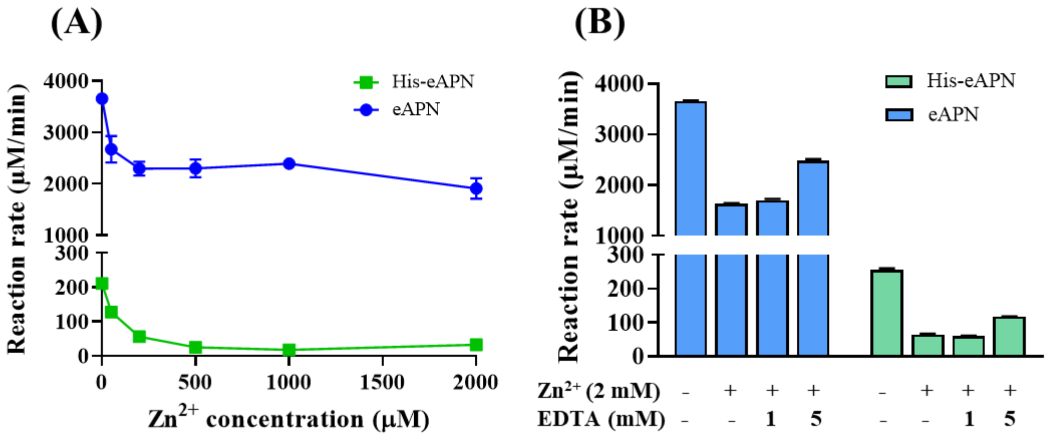

Figure S3. The effect of Zinc ions on eAPN and His-eAPN. (A) Dose-response of Zn^{2+} on enzyme activity toward L-Ala-*p*-nitroanilide. (B) The effect of 1 mM or 5 mM EDTA on enzyme activity in the presence of 2 mM Zn^{2+}.

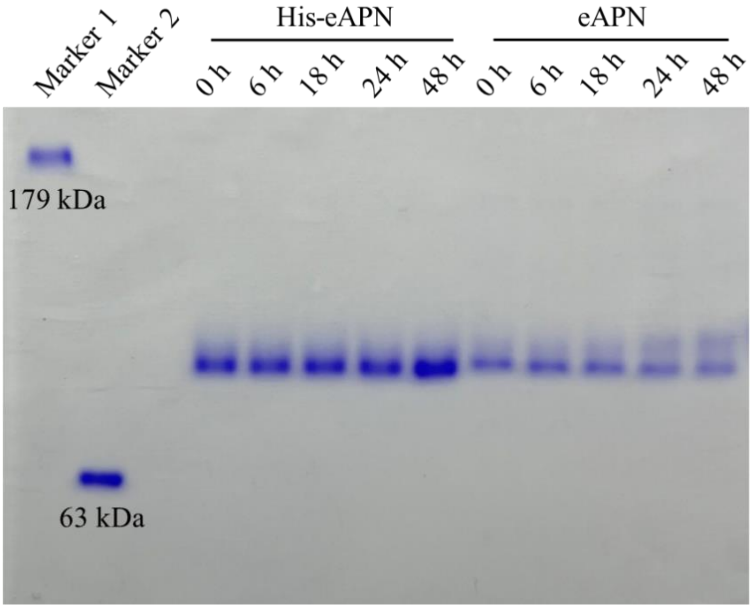

Figure S4. Native-PAGE analysis of purified eAPN and His-eAPN incubated at 37℃. Marker 1 and 2 serve as in-house protein markers, whose theoretical MW are 179 kDa and 63 kDa, respectively.

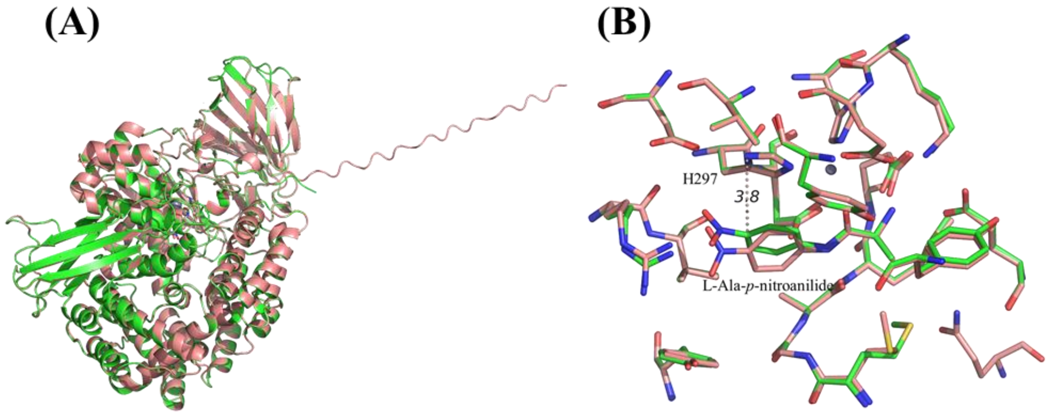

Figure S5. Superimposed Alphafold3 docking results for His-eAPN (pink) and eAPN (green). (A) overall structures; (B) active sites.

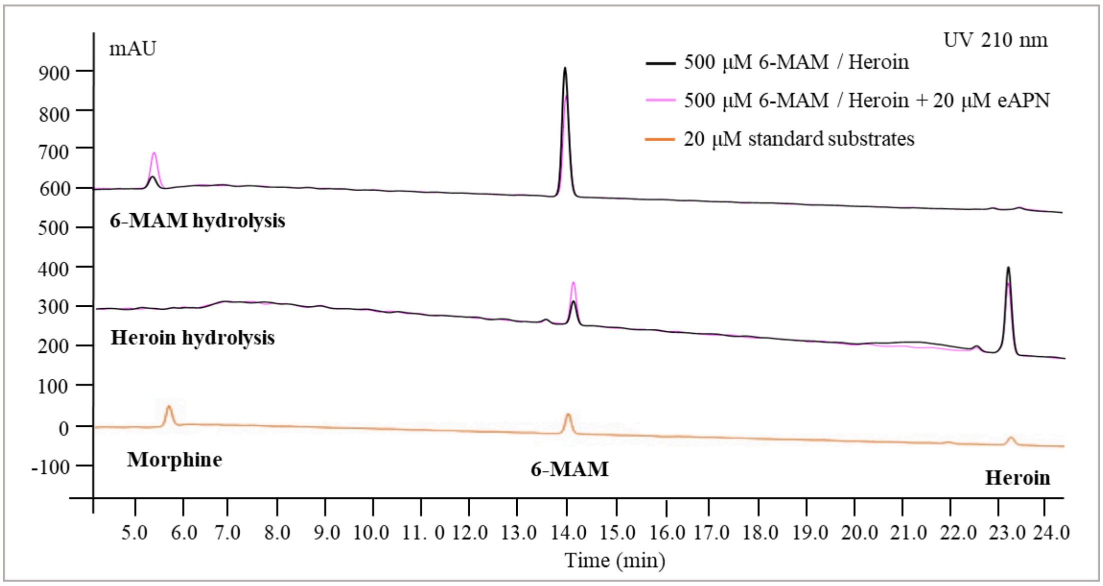

Figure S6. HPLC separation and UV detection of heroin, 6-MAM, and morphine. Pink line indicates the chromatography of 500 µM 6-MAM (24 hours) or 500 µM heroin (6 hours) incubated with 20 µM eAPN at 37°C. Black line represents the negative control (the same incubation without eAPN), and the peaks indicate the spontaneous hydrolysis during incubation. Brown line represents 20 µM standard substrates. Retention times: heroin (23.60 min), 6-MAM (13.75 min), and morphine (5.50 min).

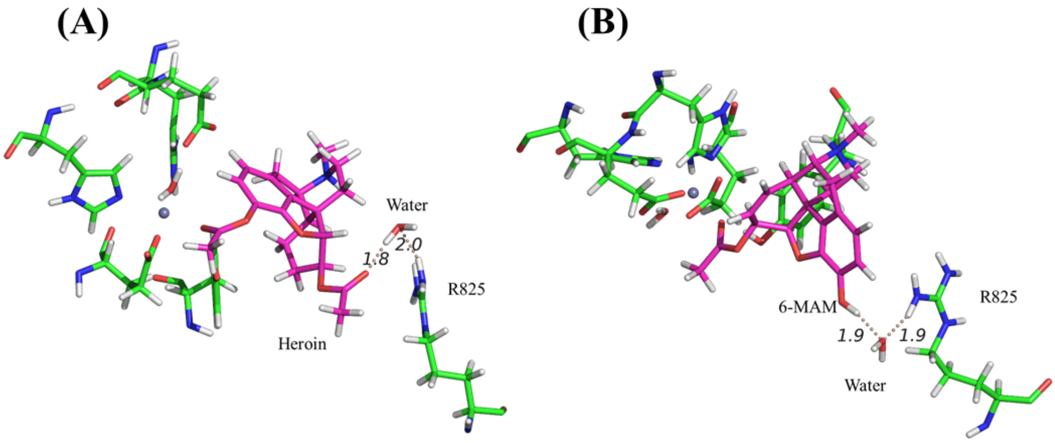

Figure S7. Hydrogen bond network in the energy-minimized ES structures for heroin (A) and 6-MAM (B) interacting with eAPN.
